# Staging and managing patients with acromegaly in clinical practice: baseline data from the SAGIT® validation study

**DOI:** 10.1007/s11102-019-00977-5

**Published:** 2019-07-23

**Authors:** Andrea Giustina, Marcello D. Bronstein, Philippe Chanson, Stephan Petersenn, Felipe F. Casanueva, Caroline Sert, Aude Houchard, Shlomo Melmed

**Affiliations:** 1 San Raffaele University Hospital Milan, 20132 Milan, Italy; 2 Hospital das Clínicas da Universidade de São Paulo, São Paulo, Brazil; 3 Assistance Publique-Hôpitaux de Paris, Hôpital de Bicêtre, Centre de Référence des Maladies Rares de l’Hypophyse (HYPO), Service d’Endocrinologie et des Maladies de la Reproduction and Univ Paris-Sud, University Paris-Saclay, Paris, Le Kremlin Bicêtre France; 4 ENDOC Centre for Endocrine Tumours, Hamburg, Germany; 5 Instituto Salud Carlos III, Santiago de Compostela University – CIBER de Fisiopatología de la Obesidad y Nutrición (CIBERobn), Santiago de Compostela, Spain; 6 Ipsen, Boulogne-Billancourt Cedex, France; 7 Cedars-Sinai Medical Center, Los Angeles, CA USA

**Keywords:** Acromegaly control, Acromegaly management, Clinician-reported outcomes, SAGIT® instrument

## Abstract

**Purpose:**

The SAGIT® instrument, designed to assist clinicians to stage acromegaly, assess treatment response and adapt patient management, was well received by endocrinologists in a pilot study. We report an interim analysis of baseline data from the validation phase.

**Methods:**

The SAGIT® validation study (ClinicalTrials.gov NCT02539927) is an international, non-interventional study. Data collection included: demographic/disease characteristics; medical/surgical histories; concomitant acromegaly treatments; investigators’ subjective evaluation of disease-control status (clinical global evaluation of disease control [CGE-DC]; controlled/not controlled/yet to be clarified) and clinical disease activity (active/not active); growth hormone (GH) and insulin-like growth factor-1 (IGF-1) levels; investigators’ therapeutic decision.

**Results:**

Of 228 patients enrolled, investigators considered disease to be controlled in 110 (48.2%), not controlled in 105 (46.1%), and yet to be clarified in 13 (5.7%) according to CGE-DC. Thirty-three patients were treatment-naïve (not controlled, *n* = 31; yet to be clarified, *n* = 2). Investigators considered 48.2% patients in the controlled and 95.2% in the not-controlled groups to have clinically active disease. In the controlled group, 29.7% of patients did not exhibit hormonal control (GH ≤ 2.5 µg/L; normalized IGF-1) and 47.3% did not have rigorous hormonal control (GH < 1.0 µg/L; normalized IGF-1) by contemporary consensus. Current acromegaly treatment was continued with no change for 91.8% of patients in the controlled and 40.0% in the not-controlled groups.

**Conclusions:**

These data highlight discrepancies between investigator-evaluated disease-control status, disease activity, hormonal control, and treatment decisions in acromegaly. Once validated, the SAGIT® instrument may assist clinicians in making active management decisions for patients with acromegaly.

**Electronic supplementary material:**

The online version of this article (10.1007/s11102-019-00977-5) contains supplementary material, which is available to authorized users.

## Introduction

Acromegaly is a multi-system disease characterized by overproduction of growth hormone (GH) and an accompanying increase in insulin-like growth factor-1 (IGF-1) levels, usually due to a GH-secreting pituitary tumor (predominantly macroadenomas). Patients exhibit characteristic morphologic changes, metabolic dysfunction, and can develop a broad range of comorbidities [1]. Acromegaly diagnosis is based on the assessment of clinical manifestations, measurement of GH and IGF-1 levels, and pituitary magnetic resonance imaging (MRI) [2]. Treatment goals for management of acromegaly include reduction of tumor size and prevention of further tumor growth, and control of excessive GH secretion and IGF-1 levels [2, 3]. Approaches to treatment include pituitary surgery, medical therapy, and radiotherapy [2, 3]. Difficulties in diagnosis (including discordant results from biochemical assays and discrepancies in signs and symptoms reported by the patient and physician) and a lack of formal disease staging using comprehensive clinical metrics, however, can impede effective management of acromegaly [4–7]. Thus, there is a need for a tool that captures biochemical, tumoral, and clinical information, to optimally diagnose, stage, and manage acromegaly.

The SAGIT® instrument has been designed by an international steering committee comprising leading experts in the management of patients with acromegaly [8]. SAGIT reflects key components associated with management of acromegaly, namely signs and symptoms [S], associated comorbidities [A], GH levels [G], IGF-1 levels [I], and tumor features [T] [8]. The SAGIT® instrument is designed to be used after initial diagnosis and during patient follow-ups to increase awareness among physicians of the need to stage the disease accurately, assess responses to treatment, and empower them to undertake rational decisions to adjust treatment when a response needs to be optimized.

The SAGIT® instrument was well accepted by endocrinologists in a pre-testing pilot study [8] that confirmed acceptability, utility, and ease of use of the instrument, and indicated its potential for distinguishing acromegaly clinical stages [8]. A clinical validation study is underway to validate scoring of the instrument and to examine its ability to discriminate between groups of patients depending on their respective levels of disease control. However, irrespective of the future performance of the SAGIT® instrument, the sizable population enrolled into the validation study could provide valuable insights into current, real-world management of patients with acromegaly.

Here, we present a pre-planned interim analysis of baseline data from patients participating in the validation phase. The aim of this interim analysis was to provide a snapshot of current practices for the management of patients with acromegaly in the real world. We report patient demographic and disease characteristics, prior surgical procedures, disease-control status determined by clinicians, and treatment decisions made. We also evaluate whether classifications of disease-control status align with disease activity, hormonal control (GH and IGF-1 levels obtained from routine hormone assays), and treatment decisions in acromegaly.

## Methods

### Study design

The SAGIT® validation study (ClinicalTrials.gov NCT02539927) is an international, non-interventional study with a 2-year follow-up period. A total of 33 centers in nine countries (Belgium, Brazil, France, Germany, Italy, Spain, the Netherlands, the UK, and the USA) participated. The longitudinal validation study has now completed and the initial data analysis is ongoing.

The trial is being conducted in accordance with the Declaration of Helsinki, the International Conference on Harmonisation, and Good Clinical Practice guidelines. Study documentation was approved by institutional review boards. Important protocol amendments occurring during the study are summarized in Supplementary Appendix S1.

### Patients

Adults (≥ 18 years) were eligible for the validation study if they had a diagnosis of acromegaly confirmed by IGF-1 levels above the age-adjusted upper limit of normal (ULN), random GH levels > 1.0 µg/L (patients with diabetes) or lack of suppression of GH levels to < 0.4 µg/L after a 75 g oral glucose load (patients without diabetes), and a pituitary adenoma visualized with MRI.

Patients were additionally required to have, in their medical records, data that would be needed to complete assessments with the SAGIT® instrument (signs and symptoms, associated comorbidities [as listed in the SAGIT® instrument], GH nadir with oral glucose tolerance test or GH random or series, IGF-1, and tumor size). Patients could be included regardless of whether they had received previous treatment for acromegaly. Patients were excluded if they had acute or severe disease (including acromegaly) that was not controlled and required intensive treatment.

Investigators subjectively evaluated baseline disease-control status of patients (clinical global evaluation of disease control [CGE-DC]), classified as ‘controlled’, ‘not controlled’, or ‘control status yet to be clarified’ (the latter was used when investigators were unsure about disease control at the specific timepoint of assessment). The study was designed to enroll balanced numbers of patients with and without disease control. The numbers of patients in the controlled and not-controlled groups were monitored at the time of the inclusion visit.

Patients receiving pegvisomant were initially enrolled. However, the ‘G’ item (GH level) of SAGIT® cannot be completed for these patients. To ensure that a sufficient number of patients with complete SAGIT® data were enrolled, the protocol was amended part-way through recruitment to exclude further enrolment of patients using pegvisomant. Recruitment was completed on September 15, 2016.

### Data collection and evaluation

Electronic case report forms (eCRFs) were completed by investigators for each patient. If some assessments were not routinely performed by investigators, the corresponding sections of the eCRFs were left blank. The following data were collected consecutively in eCRFs at baseline: demographic and disease characteristics; medical and surgical histories; information regarding concomitant treatments for acromegaly; investigators’ evaluation of the clinical activity of the disease; GH and IGF-1 levels from routine assessments; investigators’ evaluation of disease-control status (CGE-DC classification as noted above); investigators’ therapeutic decision.

Evaluation of clinical activity was assessed: the investigator recorded ‘yes’ or ‘no’ to the question, “Is your subject’s acromegaly status clinically active at this visit?”.

For hormone levels, the proportions of patients with normalized IGF-1 levels and with hormonal control (defined as GH levels ≤ 2.5/< 1.0 μg/L and normalized IGF-1 levels) were calculated from the routine assessments.

Investigators’ therapeutic decision (preferred intention) was recorded as: continue current treatment(s) with no change/no treatment initiation; intensify current treatment(s)/initiate a treatment; decrease the current treatment(s); other.

QoL at baseline was assessed using the 22-item patient-reported acromegaly QoL (AcroQoL) questionnaire [9, 10]. Data in the questionnaire provide measures of global QoL. Higher scores indicate more favorable QoL.

### Statistical analyses

It was initially estimated that at least 200 patients in total, with 100 patients in each of the CGE-DC controlled and not-controlled disease-control subgroups, were required to ensure that at least 82 patients per group had evaluable data. A higher-than-expected proportion of enrolled patients did not have sufficient data to complete all SAGIT® items at baseline (owing to the unexpectedly high inclusion of patients treated with pegvisomant, which precludes the ‘G’ [GH level] item); as such, the protocol was amended to exclude further enrolment of patients using pegvisomant and to allow enrolment to continue until the required sample size (*n* = 82 per group) was reached. A total of 82 evaluable patients per disease-control group is required to estimate an area under the receiver-operating-characteristic curve of 0.80 with 95% confidence intervals (CIs) of 0.15 width, assuming balanced group size. All consenting patients included in the study (the enrolled population) were included in the baseline analysis. Results for patients with disease-control status ‘yet to be clarified’ are not depicted in this manuscript describing baseline data, as this was a small group of patients with heterogeneous characteristics. All analyses were descriptive, with significance based on non-overlapping 95% CIs.

## Results

### Patient disposition

**Fig. 1:**
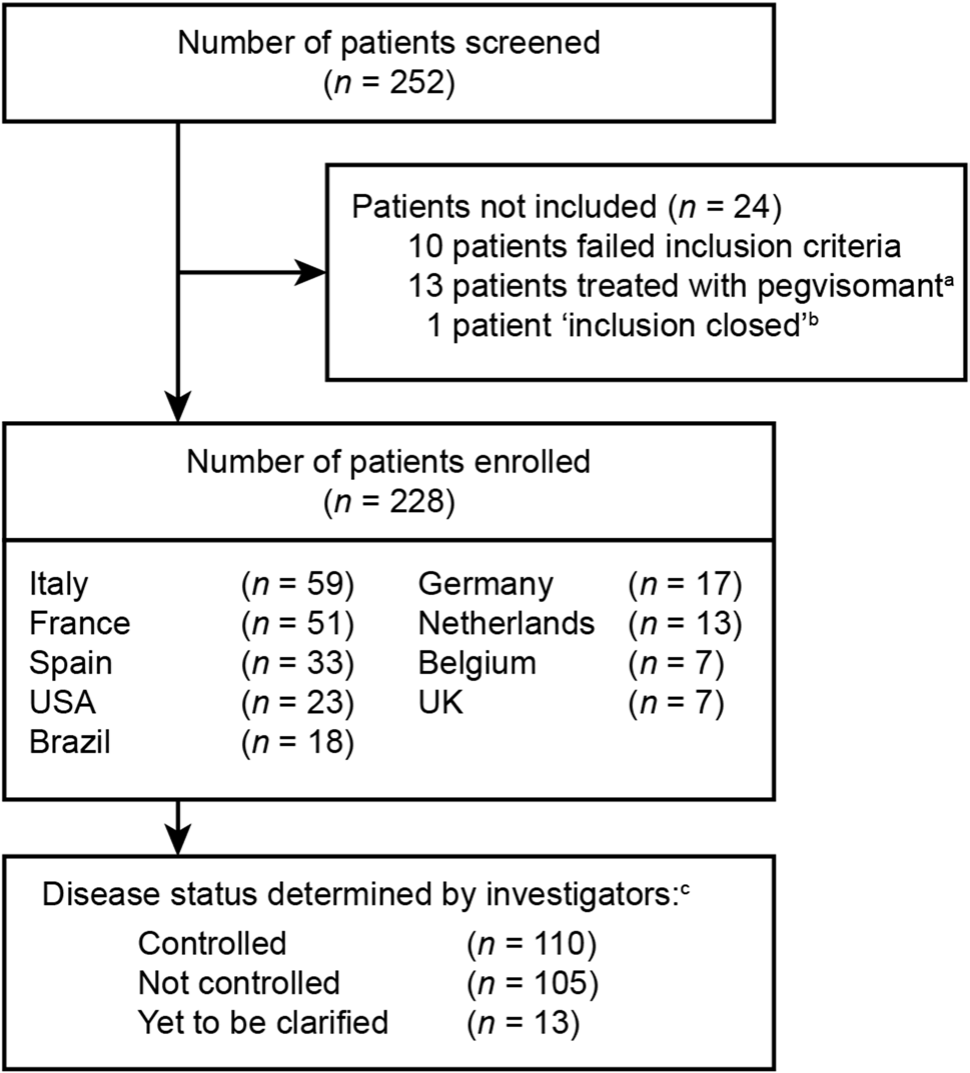
Patient disposition. ^a^A protocol amendment was introduced to exclude enrolment of patients receiving pegvisomant, to satisfy sample-size requirements for the validation of the SAGIT® instrument (the ‘G’ element of the SAGIT® instrument is not applicable to these patients). ^b^CGE-DC was not available for this patient. ^c^Investigators determined disease-control status at baseline (classified as controlled, not controlled, or status not yet clarified). *CGE-DC* clinical global evaluation of disease control

Of 252 patients screened, 228 were included in the enrolled population (Fig. 1). The population comprised 110 (48.2%) patients for whom investigators considered that disease was CGE-DC controlled, 105 (46.1%) for whom disease was considered not controlled, and 13 (5.7%) in whom disease-control status was yet to be clarified.

### Demographic and disease characteristics and treatment history

The enrolled population had a mean (SD) age of 51.6 (12.7) years, a median [range] time since diagnosis of 46.2 [0.0–390.0] months, a median (95% CI) tumor diameter of 8.0 (6.0;10.0) mm, and 47.4% of patients were male (Table 1a). The mean (SD) body mass index (BMI) of 29.1 (5.5) kg/m^2^ is rather high for an acromegaly population; five patients had a medical history of obesity (two in the CGE-DC controlled and three in the not-controlled groups). A total of 33 (14.5%) patients in the enrolled population were naïve to treatment (no previous pituitary surgery, radiotherapy, or medications for acromegaly).

**Table 1:** Baseline demographic and disease characteristics according to (a) CGE-DC categories of disease control and (b) treatment-naïve status for patients in whom disease was considered not controlled

a)	Disease-control category (CGE-DC)		Enrolled population (*n* = 228)
	Controlled (*n* = 110)	Not controlled (*n* = 105)	
Age, years	(*n* = 110)	(*n* = 105)	(*n* = 228)
Mean (SD) [95% CI], years	54.3 (11.2) [52.2;56.4]	48.8 (13.7) [46.2;51.5]	51.6 (12.7) [50.0;53.3]
< 65 years, *n* (%)	88 (80.0)	86 (81.9)	185 (81.1)
≥ 65 years, *n* (%)	22 (20.0)	19 (18.1)	43 (18.9)
Men, *n* (%)	(*n* = 110) 55 (50.0)	(*n* = 105) 48 (45.7)	(*n* = 228) 108 (47.4)
BMI, mean (SD) [95% CI], (kg/m^2^)	(*n* = 94) 28.0 (4.5) [27.1;28.9]	(*n* = 99) 30.3 (6.1) [29.1;31.5]	(*n* = 204) 29.1 (5.5) [28.4;29.9]
Time since acromegaly diagnosis	(*n* = 110)	(*n* = 105)	(*n* = 228)
Median [range], months	90.6 [6.0–390.0]	13.2 [0.0–304.8]	46.2 [0.0–390.0]
< 1 year, *n* (%)	7 (6.4)	50 (47.6)	64 (28.1)
≥ 1 year, *n* (%)	103 (93.6)	55 (52.4)	164 (71.9)
Pituitary tumor diameter^a^, (mm)	(*n* = 95)	(*n* = 96)	(*n* = 203)
Median (range) [95% CI]	3.0 (0–47) [0.0;6.0]	13.0 (0–51) [11.0;14.0]	8.0 (0–51) [6.0;10.0]
Time since MRI, median [range], months	(*n* = 101) 0.7 [0.0–183.3]	(*n* = 105) 1.2 [0.0–24.4]	(*n* = 219) 0.9 [0.0–183.3]
Treatment-naïve status Yes/no	(*n* = 110) 0/110 (100.0)	(*n* = 105) 31 (29.5)/74 (70.5)	(*n* = 228) 33 (14.5)/195 (85.5)

b)	Treatment-naïve (*n* = 31)	Not treatment-naive (*n* = 74)
Age, years	(*n* = 31)	(*n* = 74)
Mean (SD) [95% CI], years	49.2 (12.1) [44.7;53.6]	48.7 (14.4) [45.3;52.0]
< 65 years, *n* (%)	27 (87.1)	59 (79.7)
≥ 65 years, *n* (%)	4 (12.9)	15 (20.3)
Men, *n* (%)	(*n* = 31) 17 (54.8)	(*n* = 74) 31 (41.9)
BMI, mean (SD) [95% CI], (kg/m^2^)	(*n* = 29) 30.3 (5.6) [28.1;32.4]	(*n* = 70) 30.4 (6.3) [28.9;31.9]
Time since acromegaly diagnosis	(*n* = 31)	(*n* = 74)
Median [range], months	2.4 [0.0–13.2]	33.6 [0.0–304.8]
< 1 year, *n* (%)	30 (96.8)	20 (27.0)
≥ 1 year, *n* (%)	1 (3.2)	54 (73.0)
Pituitary tumor diameter^a^, (mm)	(*n* = 31)	(*n* = 65)
Median (range) [95% CI]	14.0 (5–51) [12.0;20.0]	11.0 (0–35) [8.0;14.0]
Time since MRI, median [range], months	(*n* = 31) 2 [0.1–4.1]	(*n* = 74) 1.0 [0.0–24.4]

a—Data are for all patients (enrolled population) and for groups according to CGE-DC status (controlled, not controlled); the enrolled population also included 13 patients for whom disease control status had yet to be clarified (data not shown). Supplementary Table S1 shows data by treatment-naïve status for the group in whom disease was considered not controlled

b—Data are only for the group in whom disease was considered not controlled (CGE-DC classification), as none of the patients in the controlled group were naïve to treatment. Treatment-naïve is defined as no previous surgery, radiotherapy, or medical treatments for acromegaly

*BMI* body mass index, *CGE-DC* clinical global evaluation of disease control, *CI* confidence interval, *MRI* magnetic resonance imaging, *SD* standard deviation

^a^Tumor size is from the inclusion visit (not at the time of diagnosis of acromegaly)

The most frequent prior medical or surgical events (excluding pituitary surgery) were hypertension (28.1%; 64/228), diabetes (22.8%; 52/228), and goiter (21.1%; 48/228).

The CGE-DC controlled group was significantly older than the CGE-DC not-controlled group (mean age [95% CI] 54.3 [52.2;56.4] and 48.8 [46.2;51.5] years, respectively), with lower BMI (mean [95% CI] 28.0 [27.1;28.9] and 30.3 [29.1;31.5] kg/m^2^, respectively), smaller tumors (median diameter [95% CI] 3.0 [0.0;6.0] and 13.0 [11.0;14.0] mm, respectively), and tended to have longer times since diagnosis (median [range] 90.6 [6.0–390.0] and 13.2 [0.0–304.8] months, respectively). None of the patients in the controlled group were naïve to acromegaly treatment, whereas 31/105 (29.5%) of the not-controlled group were naïve to acromegaly treatment.

Within the CGE-DC not-controlled group (between treatment-naïve and not-treatment-naïve subgroups) and between the not-controlled, treatment-naïve subgroup and the controlled group, patient demographics were similar, but some disease characteristics were different: median (range) time since diagnosis tended to be lower in the not-controlled treatment-naïve subgroup (2.4 [0.0–13.2] months), compared with not-controlled not-treatment-naive subgroup (33.6 [0.0–304.8] months), and the controlled group (90.6 [6.0–390.0] months); tumor size tended to be greater in the not-controlled treatment-naïve subgroup (median [95% CI] 14.0 [12.0;20.0] mm compared with 11.0 [8.0;14.0] mm [not-controlled not-treatment-naïve], and 3.0 [0.0;6.0] mm [controlled]).

Prior treatment for acromegaly is detailed in Table 2. Of the enrolled population, 59.6% had previously undergone pituitary surgery and 59.2% were receiving one or more medications for acromegaly at baseline. Among these patients, 110 (81.5%) were on somatostatin receptor ligands (SRLs), 28 (20.7%) were on the growth hormone receptor antagonist (GHRA) pegvisomant, 35 (25.9%) were on dopamine agonists (DAs; predominantly cabergoline), and three (2.2%) were receiving other medications. Patients receiving monotherapies at baseline included 72 (53.3%) patients on SRLs, 12 (8.9%) patients on GHRAs, and 11 (8.1%) patients on DAs. Further detail on combination therapies used at baseline can be found in Supplementary Table S1.

**Table 2:** Prior and concomitant treatments at baseline

	Disease-control category (CGE-DC)		Enrolled population (*n* = 228)
	Controlled (*n* = 110)	Not controlled (*n* = 105)	
Prior pituitary surgery	(*n* = 110) 80 (72.7)	(*n* = 105) 48 (45.7)	(*n* = 228) 136 (59.6)
Current medical treatments (mono- or combination therapies)	(*n* = 78)	(*n* = 53)	(*n* = 135)
SRLs	60 (76.9)	47 (88.7)	110 (81.5)
Lanreotide Autogel^a^	36 (60.0)	32 (68.1)	70 (63.6)
Octreotide LAR^a^	20 (33.3)	11 (23.4)	32 (29.1)
Pasireotide LAR^a^	4 (6.7)	4 (8.5)	8 (7.3)
GHRA	18 (23.1)	10 (18.9)	28 (20.7)
Pegvisomant^a^	18 (100.0)	10 (100.0)	28 (100.0)
DA	18 (23.1)	16 (30.2)	35 (25.9)
Cabergoline^a^	17 (94.4)	16 (100.0)	34 (97.1)
Bromocriptine^a^	1 (5.6)	0	1 (2.9)
Other	2 (2.6)	1 (1.9)	3 (2.2)
Monotherapies			
SRL	42 (53.8)	27 (50.9)	72 (53.3)
Lanreotide Autogel^a^	24 (57.1)	24 (88.9)	50 (69.4)
Octreotide LAR^a^	14 (33.3)	1 (3.7)	16 (22.2)
Pasireotide LAR^a^	4 (9.5)	2 (7.4)	6 (8.3)
GHRA	10 (12.8)	2 (3.8)	12 (8.9)
Pegvisomant^a^	10 (100.0)	2 (100.0)	12 (100.0)
DA	7 (9.0)	3 (5.7)	11 (8.1)
Cabergoline^a^	7 (100.0)	3 (100.0)	11 (100.0)
Combination therapy			
SRL with pegvisomant	8 (10.3)	7 (13.2)	15 (11.1)
SRL with DA	9 (11.5)	12 (22.6)	21 (15.6)
SRL, DAs with pegvisomant	2 (2.6)	0	2 (1.5)

Data are for all patients (enrolled population) and for groups according to CGE-DC status (controlled, not controlled); the enrolled population also included 13 patients for whom disease control status had yet to be clarified (data not shown). Data are number (%) of patients in the enrolled population (or subpopulation thereof) or ^a^of patients receiving a medication in the corresponding therapeutic class

*CGE-DC* clinical global evaluation of disease control, *DA* dopamine agonist, *GHRA* growth hormone receptor antagonist, *LAR* long-acting release, *SRL* somatostatin receptor ligand

### Assessments of clinically active disease status

Investigators recorded that 70.2% (160/228) of patients in the enrolled population had clinically active disease (as per investigator subjective evaluation of disease activity) at the baseline visit. Investigators considered 48.2% (53/110) of the CGE-DC controlled group to have clinically active disease. Almost all patients (95.2%; 100/105) in the CGE-DC not-controlled group were considered to have clinically active acromegaly, with similar proportions in the treatment-naïve (93.5% [29/31]) and not-treatment-naïve (95.9% [71/74]) subgroups.

### Hormone levels

The CGE-DC controlled group had significantly lower GH and IGF-1 levels at baseline than the not-controlled group (Table 3a). Within the CGE-DC not-controlled group, GH levels tended to be higher in patients in the treatment-naïve subgroup compared with patients in the not-treatment-naïve subgroup, as well as the proportion of patients with IGF-1 levels ≥ 2 × ULN (Table 3b).

**Table 3:** Baseline GH and IGF-1 levels according to (a) CGE-DC categories of disease control and (b) treatment-naïve status for patients in whom disease was considered not controlled

a)	Disease-control category (CGE-DC)		
	Controlled (*n* = 110)	Not controlled (*n* = 105)	Enrolled population (*n* = 228)
Random or series GH levels in µg/L^a^	(*n* = 75)	(*n* = 52)	(*n* = 135)
Mean (SD) [95% CI]	1.06 (1.92) [0.62;1.50]	5.08 (8.58) [2.69;7.46]	2.71 (5.88) [1.71;3.71]
≤ 1.0, *n* (%)	52 (69.3)	9 (17.3)	66 (48.9)
> 1.0 to < 2.5, *n* (%)	18 (24.0)	16 (30.8)	34 (25.2)
≥ 2.5 to < 5.0, *n* (%)	4 (5.3)	12 (23.1)	18 (13.3)
≥ 5 to < 10, *n* (%)	0	10 (19.2)	10 (7.4)
≥ 10, *n* (%)	1 (1.3)	5 (9.6)	7 (5.2)
GH nadir in µg/L^a^	(*n* = 15)	(*n* = 39)	(*n* = 59)
Mean (SD) [95% CI]	0.49 (0.63) [0.14;0.84]	10.37 (16.51) [5.02;15.72]	7.34 (14.16) [3.65;11.03]
≤ 0.4, *n* (%)	9 (60.0)	1 (2.6)	10 (16.9)
> 0.4 to < 1.0, *n* (%)	4 (26.7)	2 (5.1)	8 (13.6)
≥ 1.0 to < 2.5, *n* (%)	2 (13.3)	11 (28.2)	14 (23.7)
≥ 2.5 to < 5, *n* (%)	0	7 (17.9)	8 (13.6)
≥ 5, *n* (%)	0	18 (46.2)	19 (32.2)
IGF-1 levels in × ULN	(*n* = 110)	(*n* = 104)	(*n* = 227)
Mean (SD) [95% CI]	0.79 (0.25) [0.74;0.84]	2.19 (1.16) [1.96;2.41]	1.47 (1.06) [1.33;1.61]
≤ 1.0, *n* (%)	90 (81.8)	9 (8.7)	101 (44.5)
< 1.3, *n* (%)	107 (97.3)	26 (25.0)	141 (62.1)
> 1.0 to < 1.3, *n* (%)	17 (15.5)	17 (16.3)	40 (17.6)
≥ 1.3 to < 2.0, *n* (%)	3 (2.7)	27 (26.0)	33 (14.5)
≥ 2.0, *n* (%)	0	51 (49.0)	53 (23.3)

b)	Treatment-naïve (*n* = 31)	Not treatment-naïve (*n* = 74)
Random or series GH levels in µg/L^a^	(*n* = 6)	(*n* = 46)
Mean (SD) [95% CI]	6.08 (4.45) [1.41;10.75]	4.94 (9.01) [2.27;7.62]
≤ 1.0, *n* (%)	0	9 (19.6)
> 1.0 to < 2.5, *n* (%)	2 (33.3)	14 (30.4)
≥ 2.5 to < 5.0, *n* (%)	1 (16.7)	11 (23.9)
≥ 5 to < 10, *n* (%)	2 (33.3)	8 (17.4)
≥ 10, *n* (%)	1 (16.7)	4 (8.7)
GH nadir in µg/L^a^	(*n* = 25)	(*n* = 14)
Mean (SD) [95% CI]	13.63 (19.57) [5.56;21.71]	4.55 (5.69) [1.26;7.83]
≤ 0.4, *n* (%)	0	1 (7.1)
> 0.4 to < 1.0, *n* (%)	1 (4.0)	1 (7.1)
≥ 1.0 to < 2.5, *n* (%)	4 (16.0)	7 (50.0)
≥ 2.5 to < 5, *n* (%)	5 (20.0)	2 (14.3)
≥ 5, *n* (%)	15 (60.0)	3 (21.4)
IGF-1 levels in × ULN	(*n* = 31)	(*n* = 73)
Mean (SD) [95% CI]	3.29 (0.97) [2.94;3.65]	1.72 (0.88) [1.51;1.92]
≤ 1.0, *n* (%)	0	9 (12.3)
< 1.3, *n* (%)	0	26 (35.6)
> 1.0 to < 1.3, *n* (%)	0	17 (23.3)
≥ 1.3 to < 2.0, *n* (%)	2 (6.5)	25 (34.2)
≥ 2.0, *n* (%)	29 (93.5)	22 (30.1)

a—Data are for all patients (enrolled population) and for groups according to CGE-DC status (controlled, not controlled); the enrolled population also included 13 patients for whom disease control status had yet to be clarified (data not shown)

b—Data are shown by treatment-naïve status for the group in whom disease was considered not controlled but not for the subgroup in whom disease was considered controlled, as all patients in the latter subgroup had received previous treatment. Treatment-naïve is defined as no previous surgery, radiotherapy, or medical treatments for acromegaly

*CGE-DC* clinical global evaluation of disease control, *CI* confidence interval, *GH* growth hormone, *IGF-1* insulin-like growth factor-1, *SD* standard deviation, *ULN* upper limit of normal

^a^GH data exclude patients receiving pegvisomant

#### GH levels

Mean [95% CI] GH nadir was 13.63 [5.56;21.71] µg/L in the treatment-naïve (*n* = 25) and 4.55 [1.26;7.83] µg/L in the not-treatment-naïve (*n* = 14) subgroups; mean [95% CI] random or series GH levels were 6.08 [1.41;10.75] µg/L in the treatment-naïve subgroup (*n* = 6) and 4.94 [2.27;7.62] µg/L in the not-treatment-naïve subgroup (*n* = 46); however, due to the small and unbalanced sample sizes, no conclusions could be drawn.

#### IGF-1 levels

Similar proportions of patients in the CGE-DC controlled (15.5%) and CGE-DC not-controlled (16.3%) groups had IGF-1 levels > 1.0 to < 1.3 × ULN (Table 3a), and 26.0% of patients in the CGE-DC not-controlled group had IGF-1 levels between ≥ 1.3 and < 2.0 × ULN. In the CGE-DC not-controlled not-treatment-naïve subgroup, 34.2% of patients had IGF-1 levels between ≥ 1.3 and < 2.0 × ULN (Table 3b). Almost all treatment-naïve patients (93.5%) had IGF-1 ≥ 2.0 × ULN, compared with 30.1% of not-treatment-naïve patients.

#### Hormonal control

**Fig. 2:**
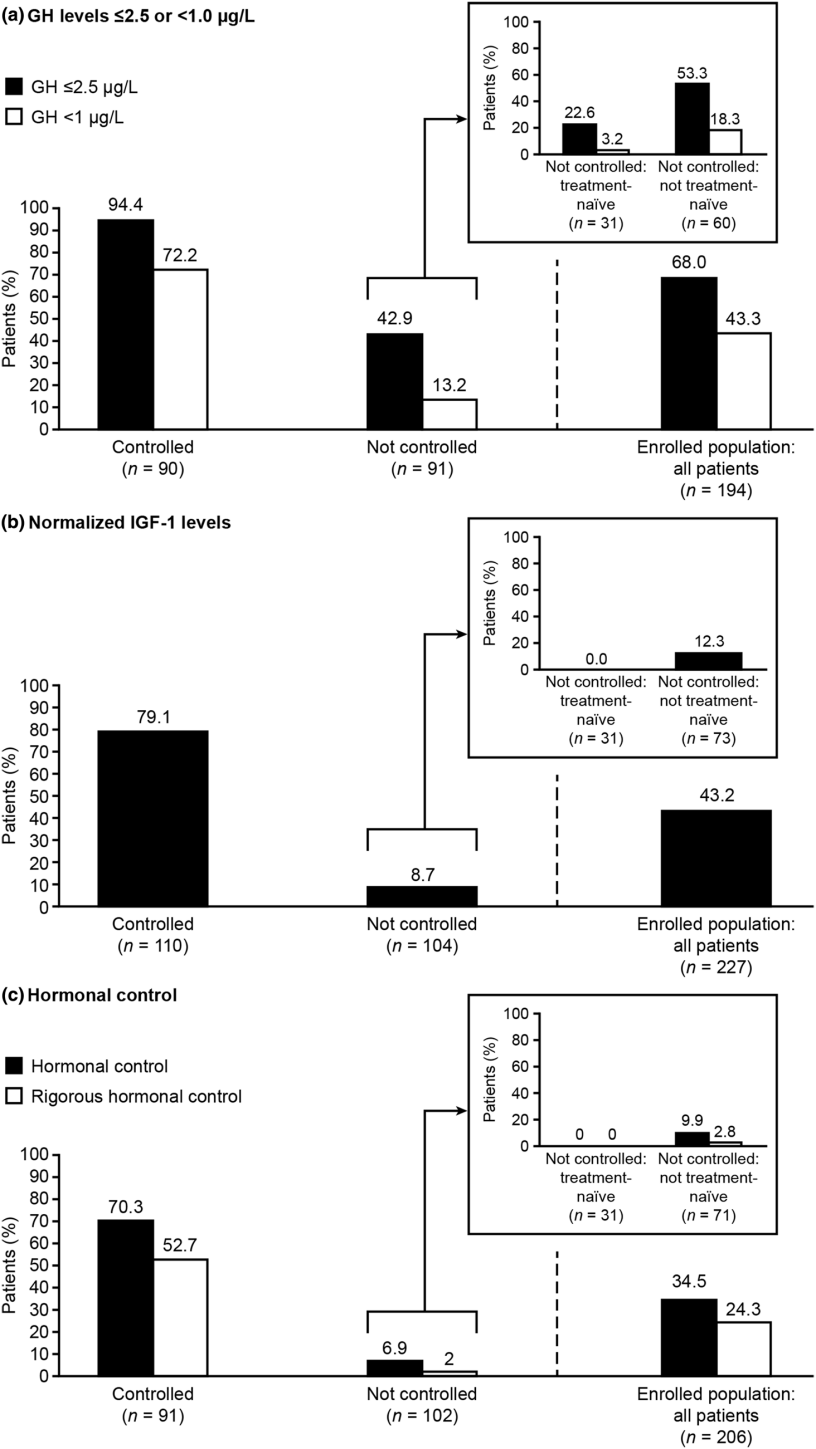
Patients with GH levels ≤ 2.5/< 1.0 µg/L and/or normalized IGF-1 levels according to CGE-DC categories of disease control. Hormone control defined as GH levels ≤ 2.5 µg/L and normalized IGF-1 levels; rigorous hormonal control defined as GH levels ≤ 1.0 µg/L and normalized IGF-1 levels. Hormone control was derived for some patients based on uncontrolled IGF-1 levels alone. GH data exclude patients receiving pegvisomant. Results are for all patients (enrolled population) and for groups according to CGE-DC status (controlled, not controlled); the enrolled population also included 13 patients for whom disease control status had yet to be clarified (data not shown). Data are shown by treatment-naïve status as an insert for the group in whom disease was considered not controlled; there are no corresponding data for the group in whom disease was considered controlled, as all patients had received previous treatment. Treatment-naïve is defined as no previous surgery, radiotherapy, or medical treatments for acromegaly. *CGE-DC* clinical global evaluation of disease control, *GH* growth hormone, *IGF-1* insulin-like growth factor-1

The proportions of patients with GH levels ≤ 2.5 or < 1.0 μg/L, normalized IGF-1, and hormonal control (GH levels ≤ 2.5/< 1.0 μg/L and normalized IGF-1 levels) at baseline are shown in Fig. 2a–c. In the CGE-DC controlled group, 29.7% of patients did not exhibit hormonal control at baseline (GH levels ≤ 2.5 µg/L and normalized IGF-1 levels), and 47.3% did not have rigorous hormonal control (GH levels < 1.0 µg/L and normalized IGF-1 levels) (Fig. 2c). In the CGE-DC not-controlled group, 93.1% of patients did not have hormonal control (GH levels ≤ 2.5 µg/L and normalized IGF-1 levels) and 98.0% did not achieve rigorous hormonal control (GH levels < 1.0 µg/L and normalized IGF-1 levels) (Fig. 2c).

### Investigators’ therapeutic decisions

**Fig. 3:**
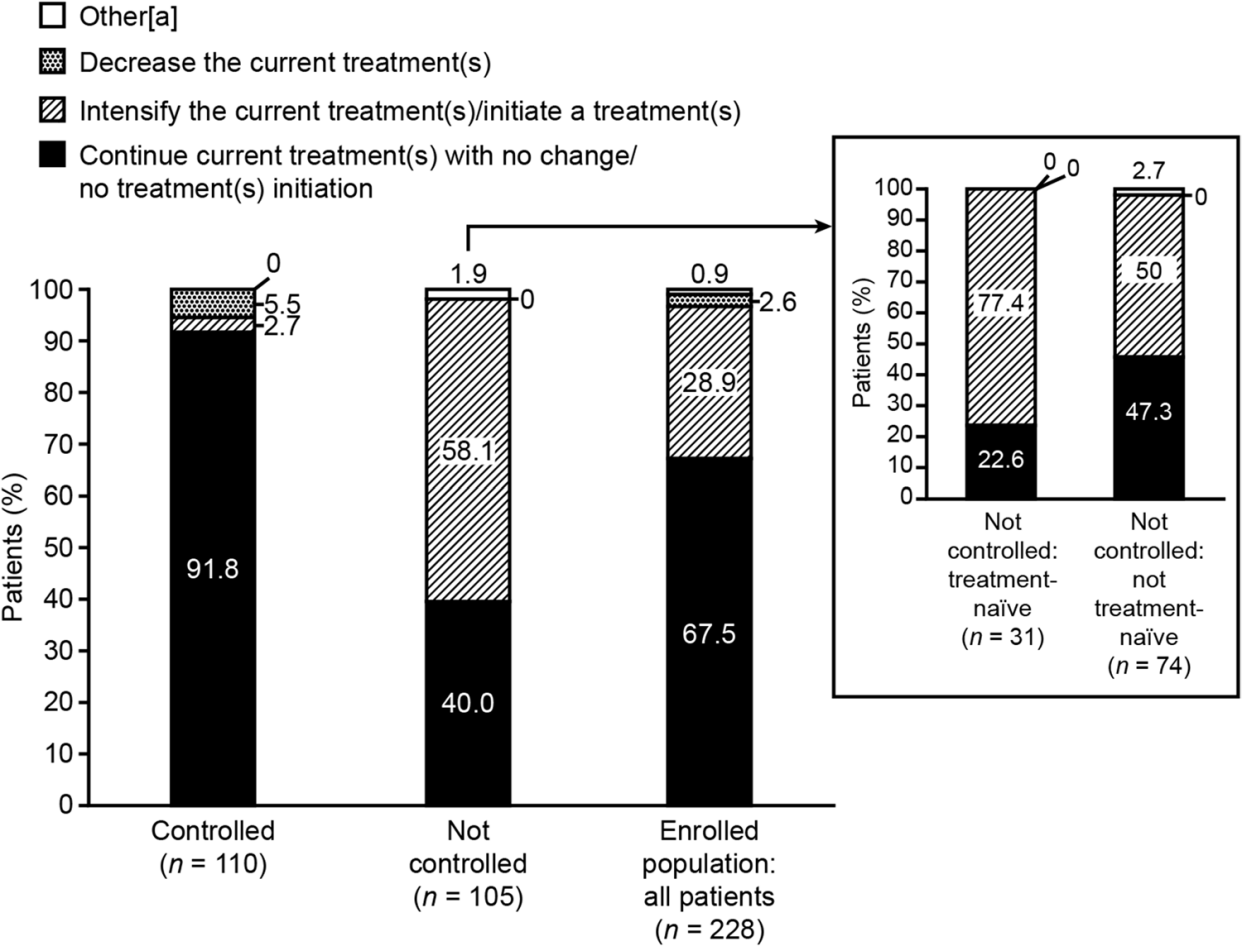
Investigators’ therapeutic decisions according to CGE-DC categories of disease control. Results are for all patients (enrolled population) and for groups according to CGE-DC status (controlled, not controlled); the enrolled population also included 13 patients for whom disease control status had yet to be clarified (data not shown). ^a^Other investigator therapeutic attitudes at baseline were ‘under evaluation for neurosurgery’ and ‘will initiate a treatment 3 months following surgery’. *CGE-DC* clinical global evaluation of disease control

Investigators continued acromegaly treatment with no change/did not initiate a treatment at baseline in 67.5% of the enrolled population (Fig. 3). Acromegaly treatment was unchanged in 91.8% of the CGE-DC controlled group. Investigators did not change acromegaly treatment at the baseline visit for 40.0% of those for whom disease had been classified as CGE-DC not controlled. This proportion corresponded to treatment unchanged in 22.6% of treatment-naïve patients (seven patients) and 47.3% of not-treatment-naïve patients (35 patients). In 5.5% (6/110) of patients in the CGE-DC controlled group, physicians chose to decrease the current acromegaly treatment.

When the therapeutic decision was examined by the country where the patients were recruited, the number of patients in each group was too low to draw firm conclusions; however, potential geographic differences were apparent in the proportion of patients for whom treatment was left unchanged (Supplementary Tables S2 and S3). In the CGE-DC not-controlled subgroup, the countries with a slightly greater proportion of patients whose treatment was left unchanged were Belgium, Brazil, Germany, and the UK. On average, in the CGE-DC not-controlled subgroup, treatment was intensified or a new treatment was initiated in 58.1% of patients (Supplementary Table S3).

### QoL

**Fig. 4:**
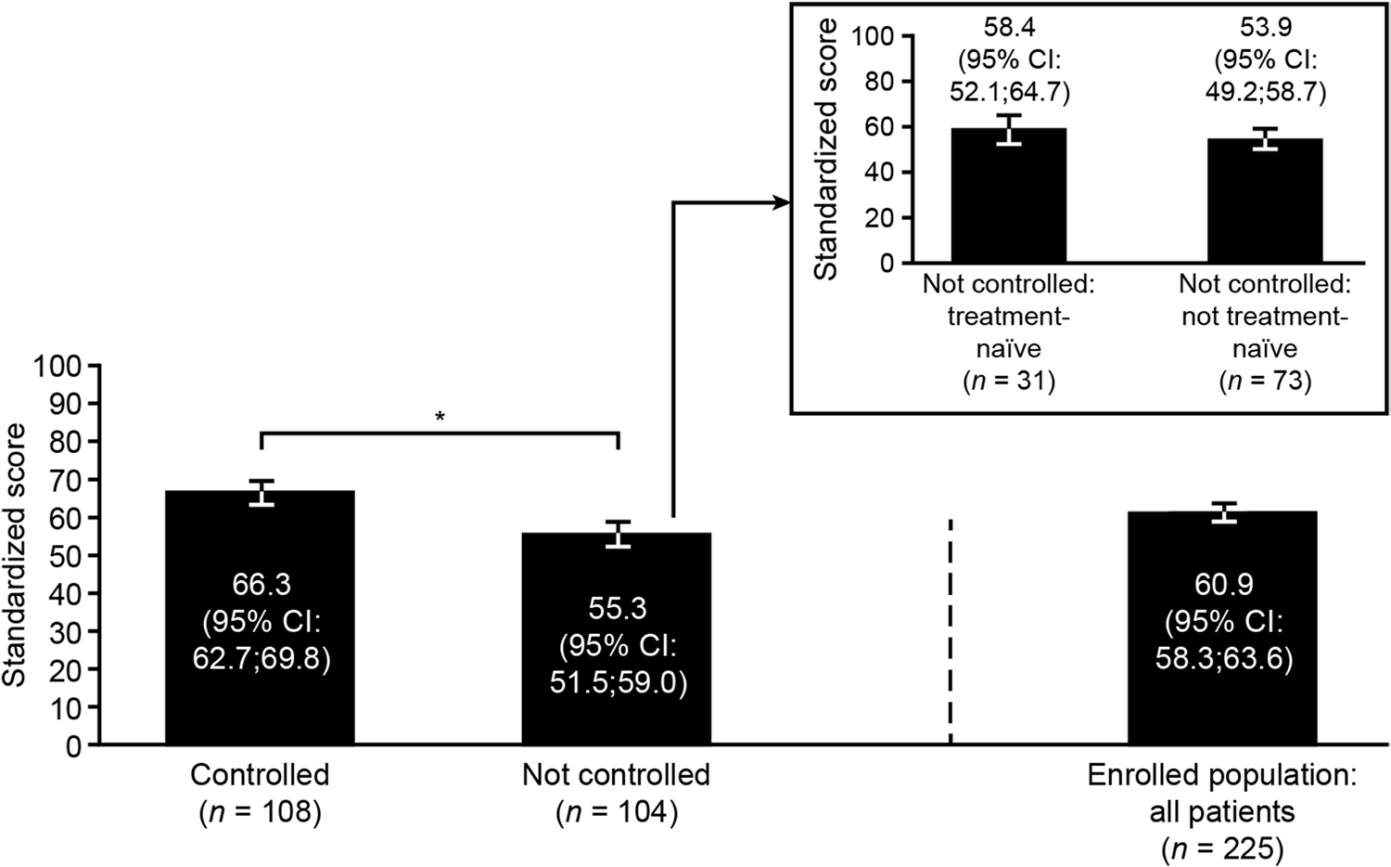
AcroQoL questionnaire global scores at baseline according to CGE-DC categories of disease control. Data are presented as the mean and 95% CIs for the mean (error bars) for all patients (enrolled population) and for subgroups according to CGE-DC categories (controlled, not controlled); the enrolled population also included 13 patients for whom disease control status had yet to be clarified (data not shown). Data are shown by treatment-naïve status as an insert for the group in whom disease was considered not controlled; there are no corresponding data for the group in whom disease was considered controlled, as all patients had received previous treatment. Treatment-naïve is defined as no previous surgery, radiotherapy, or medical treatments for acromegaly. Higher scores indicate better quality of life. *Significantly different based on non-overlapping 95% CIs. *AcroQoL* acromegaly quality of life questionnaire, *CI* confidence interval, *CGE-DC* clinical global evaluation of disease control

Compared with the CGE-DC not-controlled group, the CGE-DC controlled group had a significantly better AcroQoL global (66.3 [62.7, 69.8] vs. 55.3 [51.5, 59.0]) score at baseline (Fig. 4). No significant differences were apparent between the treatment-naïve and not-treatment-naïve subgroups within the CGE-DC not-controlled group.

## Discussion

The SAGIT® instrument has been designed to assist clinicians in staging acromegaly, assessing treatment response and adapting patient management appropriately and was well received by endocrinologists in a pilot study [8]. Here, the pre-planned interim analysis of baseline data from patients participating in the validation study, an international, non-interventional study with a 2-year follow-up period, were reported. In this sizable cohort of patients with acromegaly (*n* = 228; 110 CGE-DC controlled, 105 not controlled, and 13 yet to be clarified), the majority of patients considered as controlled by clinicians exhibited hormonal control; however, 29.7% did not have hormonal control. In 91.8% of the CGE-DC controlled group (and therefore including at least the majority of the 29.7% without hormonal control), no modification was made to baseline treatment. In the case of the CGE-DC not controlled group, the majority of patients did not exhibit either hormonal control (93.1%) or rigorous hormonal control (98.0%), but despite this, no modification to baseline treatment was made for 40% of this group. Importantly, it should be noted that, in the not-controlled group, clinicians initiated treatment in “only” 76% of patients in the treatment-naïve subgroup and made no modification to treatment in 46% of patients in the not-treatment-naïve subgroup.

In almost all patients with disease considered not controlled, investigators answered that their patient had clinically active acromegaly. Interestingly, investigators indicated that clinically active acromegaly was also present in 48% of patients considered controlled. Control status was determined based upon a subjective global evaluation, measured by CGE-DC to assess the disease control at the inclusion visit. These findings also illustrate that investigator opinion of “control” may differ quite widely, with different aspects given greater priority by different caregivers. For example, patients may be considered as controlled if only IGF-1 is controlled, if both GH and IGF-1 are controlled, if hormonal control and symptom control are achieved, or if only symptom control is demonstrated despite slightly higher IGF-1 levels (between > 1.3 and < 2 × ULN). Some investigators may only consider their patient to be not controlled if symptoms persist. Importantly, rigorous definitions of hormonal control may not be uniformly applied in clinical practice. In addition, discrepancies between GH and IGF-1 have been well described in the literature [5, 6], and might be attributed to polymorphisms of the GH receptor [11]. It is imperative that patients who are not controlled are identified and treated, as they are at increased risk of morbidity and mortality due to negative effects of raised GH and IGF-1 levels. The current consensus for cure and remission of acromegaly is based upon biochemical control e.g., age-adjusted normal range of IGF-1 and GH < 1.0 µg/L (random GH measurement) [12, 13]. SAGIT® staging will examine not only biochemical parameters, but also signs and symptoms, associated comorbidities, and tumor features, and thus provide the possibility to better address the patient regarding disease control.

Many practical considerations may influence why no treatment was initiated, changed, or increased in dose in such a sizable proportion of patients considered not controlled, including those who were naïve to treatment. For some of this latter subgroup there may have been a lack of available or reimbursed medication, and patient preference may also have had an influence. In addition, IGF-1 levels may not have been regarded as sufficiently elevated to warrant treatment. The lack of treatment initiation, change, or increase in dose could also be attributed to clinical inertia (i.e. a ‘watch and wait’ attitude), particularly if, for example, patients require intensification of treatment from a monthly SRL injection to a daily GHRA injection. A survey-based analysis of the patients included in the German Acromegaly Registry Database demonstrated that, in most cases, reasons for longstanding active acromegaly were the patient’s reluctance to comply with therapeutic recommendations (escalate/step up treatment), variable IGF-1 levels, and patient non-compliance with medical therapy [14]. Further confusion can be caused by discordant clinical and biochemical profiles, which can hinder decision-making processes [2]. In terms of geographical differences, the size of the treatment center and the number of patients receiving treatment can affect therapeutic choices. In addition, country-specific treatment preferences may exist. Indeed, a preference for SRLs as primary medical treatment of acromegaly has been observed in Europe, whereas pegvisomant is more commonly used as a first-line treatment in the USA [15]. A recent survey exploring acromegaly management in North Africa and the Middle East found that the greatest barriers to optimal treatment of the disease were high cost of medications and lack of physician awareness, both of which were reported by over a third of physicians surveyed [16].

There are currently many challenges in the clinical management of acromegaly following diagnosis. Ideally, monitoring and management of patients with acromegaly would be performed in a pituitary tumor center of excellence, where experienced interdisciplinary teams can provide timely and effective management of the disease [17]. However, access to such facilities may be limited for some patients and in certain regions. Consensus criteria for disease control have their place, but clinicians need more practical tools to assist in assessment and monitoring of disease stage and progression. Besides SAGIT®, ACRODAT® is one initiative attempting to address these needs. It is a software tool using IGF-I and tumor status to support routine clinical decision-making for patients with moderate or severe disease activity [18]. AcroVoice is a two-phase (qualitative and quantitative) study designed to assess the relevance and relative importance of ACRODAT® parameters from the patient’s perspective. A prospective study is planned to evaluate whether patients monitored by ACRODAT® will benefit in terms of improved treatment outcome. Interestingly, favorable factors associated with disease control, including older age, smaller tumors, and longer time from diagnosis to treatment, are consistent with reported personalized markers of control [19, 20].

The SAGIT® validation study may provide a definitive validation of the instrument, as well as longitudinal data that are, by definition, not available in this baseline snapshot: changes in disease-control status and ‘clinically active’ status from one visit to the next. It may also become clear how the status of patients with control status yet to be clarified evolves.

Non-interventional designs have inherent limitations, but this is an appropriate choice of design given the purpose of the study. Certain aspects of data collection were performed retrospectively, and information on medical treatment prior to surgery was not collected. GH and IGF-1 measurements relied on local laboratory testing rather than centralized assessment. It is not standard practice to evaluate data according to treatment center and although comparison of baseline data between countries would be of great interest, the study was not powered for such analysis and the sample sizes per country were too small for a valid comparison. Nevertheless, there are few studies available on patients with acromegaly and the baseline findings reported here are an important window into the real-life current clinical situation.

In summary, this interim analysis of baseline data from a study using a sizable cohort of patients with acromegaly shows that the majority of patients considered to be CGE-DC controlled by clinicians exhibited hormonal control, and no modification was made to baseline treatment in almost all cases. The majority of patients considered as CGE-DC not controlled by clinicians did not exhibit hormonal control and, in many cases, no modification to baseline treatment was made. A note of caution should be applied, however, as rigorous definitions of hormonal control may not be uniformly applied in clinical practice. Importantly, the baseline data from this study also show that some physicians do not initiate or escalate treatment even if patients are not controlled (naïve or not). Clinicians did initiate treatment in over three-quarters of patients in the not-controlled treatment-naïve subgroup, and made no modification to treatment in nearly half of patients in the not-controlled not-treatment-naïve subgroup. It should be noted that changes to treatment suggested by investigators are not always implemented due to several reasons. Interestingly, investigators indicated that clinically active acromegaly was also present in 48% of patients considered CGE-DC controlled. These clinical, standard-of-care practice findings affirm the need for better monitoring and management of patients with acromegaly.

## Electronic supplementary material

Below is the link to the electronic supplementary material.

Supplementary material 1 (DOCX 44 kb)

## Data Availability

Where patient data can be anonymized, Ipsen will share all individual participant data that underlie the results reported in this article with qualified researchers who provide a valid research question. Study documents, such as the study protocol and clinical study report, are not always available. Proposals should be submitted to DataSharing@Ipsen.com and will be assessed by a scientific review board. Data are available beginning 6 months and ending 5 years after publication; after this time, only raw data may be available.
