# Supplementary material for: Staging and managing patients with acromegaly in clinical practice: baseline data from the SAGIT® validation study
Source: Pituitary. 2019 Jul 23;22(5):476–87. doi: 10.1007/s11102-019-00977-5 (PMC6728296; doi:10.1007/s11102-019-00977-5)
Supplement: Supplementary file 1 — Supplementary material 1 (DOCX 44 kb) [file 11102_2019_977_MOESM1_ESM.docx]

**Supplementary material and additional data**

**Staging and managing patients with acromegaly in clinical practice: baseline data from the SAGIT® validation study**

Andrea Giustina, Marcello D Bronstein, Philippe Chanson, Stephan Petersenn, Felipe F Casanueva, Caroline Sert, Aude Houchard, Shlomo Melmed

Journal: *Pituitary*

**Corresponding author:** Prof. Andrea Giustina, San Raffaele University Hospital Milan, 20132, Milan, Italy

Email: giustina.andrea@hsr.it

**Appendix S1**

*Important protocol amendments*

Protocol amendment #2 was dated 15 June 2016. Reasons for the amendment were as follows:

- The primary endpoint of the SAGIT® validation study is based on SAGIT® instrument full completion at baseline (all five items). For patients treated by Pegvisomant, the item “G”(corresponding to GH) is not relevant to be completed (Pegvisomant is an antagonist of GH receptor). As a consequence, SAGIT® instrument at baseline cannot be completed for these patients for the item “G” and this could create a bias for the analysis of the primary endpoint. As of today, 10 patients are treated by Pegvisomant in each of the group (controlled, not controlled) and this is representative of clinical practice (confirmed by the Swedish registry patients, Ipsen data on file). For those reasons and as per experts’ recommendation, the enrollment of patients treated by Pegvisomant will not be recommended anymore.

**Appendix S2**

*SAGIT® Investigator Group*

**Belgium:** A. Beckers; **Brazil:** J. Abucham, M. D. Bronstein, M.R. Gadelha; **Denmark:** U. Feldt‑Rasmussen, P. Løgstrup Poulsen; **France:** F. Borson-Chazot, P. Caron, F. Castinetti, P. Chanson; **Germany:** M. Droste, S. Petersenn, N. Unger; **Italy:** F. Bogazzi, L. De Marinis, R. Pivo Nello, T. Porcelli, R. Vettor; **Netherlands:** N. Biermasz, A. Hermus, R. Netea- Maier, S. Neggers; **Spain:** C. Alvarez Escola, F. Casanueva, J. Mesa, E. Venegas; **UK:** P. Abraham, M. Gurnell, N. Karavitaki, A. Rees; **US**: O. Cooper, M.B. Gordon, A.G. Ioachimescu, L. Kennedy.

**Table S1**. Concomitant combination therapies at baseline

|  | **Controlled (*n* = 110)** | **Not controlled (*n* = 105)** | **Enrolled population (*n* = 228)** |
| --- | --- | --- | --- |
| Patients currently receiving medication for acromegaly, *n* (%) | 78 (70.9) | 53 (50.5) | 135 (59.2) |
| Patient under DA and GHRA,^a^ *n* (%) | 2 (2.6) | 1 (1.9) | 3 (2.2) |
| Patient under CAB and PEG,^b^ *n* (%) | 2 (100.0) | 1 (100.0) | 3 (100.0) |
| Patient currently receiving a combination of acromegalic medications including SRL, n (%) | 18 (16.4) | 20 (19.0) | 38 (16.7) |
| Patient under SRL and DA,^a^ *n* (%) | 11 (14.1) | 12 (22.6) | 23 (17.0) |
| Patient under LAN and CAB,^b^ *n* (%) | 7 (63.6) | 4 (33.3) | 11 (47.8) |
| Patient under OCT and CAB,^b^ *n* (%) | 3 (27.3) | 6 (50.0) | 9 (39.1) |
| Patient under OCT and BRO,^b^ *n* (%) | 1 (9.1) | 0 | 1 (4.3) |
| Patient under PAS and CAB,^b^ *n* (%) | 0 | 2 (16.7) | 2 (8.7) |
| Patient under SRL and GHRA,^a^ *n* (%) | 8 (10.3) | 7 (13.2) | 15 (11.1) |
| Patient under LAN and PEG,^b^ *n* (%) | 6 (75.0) | 3 (42.9) | 9 (60.0) |
| Patient under OCT and PEG,^b^ *n* (%) | 2 (25.0) | 4 (57.1) | 6 (40.0) |
| Patient under SRL and OTHER therapy^a^ | 1 (1.3) | 1 (1.9) | 2 (1.5) |
| Patient under LAN and OTHER therapy,^b^ *n* (%) | 1 (100.0) | 1 (100.0) | 2 (100.0) |
| Patient under SRL and DA and GHRA,^a^ *n* (%) | 2 (2.6) | 0 | 2 (1.5) |
| Patient under LAN and CAB and PEG,^b^ *n* (%) | 2 (100.0) |  | 2 (100.0) |

Data are for all patients (enrolled population) and for groups according to CGE-DC status (controlled, not controlled); the enrolled population also included 13 patients for whom disease control status had yet to be clarified (data not shown).

^a^Percentages are based on the number of patients currently receiving a medication for acromegaly.

^b^Percentages are based on the number of patients currently receiving a medication in the corresponding combination.

DA, dopamine agonist (i.e. CAB: cabergoline and BRO: bromocriptine); GHRA, growth hormone receptor antagonist (i.e. PEG: pegvisomant); SRL, somatostatin receptor ligand (i.e. LAN: lanreotide Autogel; OCT: octreotide LAR; and PAS: pasireotide LAR).

**Table S2.** Investigator therapeutic attitude at baseline by country – controlled patients

| **Investigator therapeutic attitude at baseline** | **Belgium (*n* = 4)** | **Brazil (*n* = 2)** | **France (*n* = 23)** | **Germany (*n* = 6)** | **Italy (*n* = 33)** | **Spain (*n* = 25)** | **UK (*n* = 2)** | **USA (*n* = 15)** | **Total (*n* = 110)** |
| --- | --- | --- | --- | --- | --- | --- | --- | --- | --- |
| Continue the current treatment (s) with no change/no treatment(s) initiation, *n* (%) | 4 (100) | 2 (100) | 21 (91.3) | 6 (100) | 29 (87.9) | 23 (92.0) | 2 (100) | 14 (93.3) | 101 (91.8) |
| Intensify the current treatment(s)/initiate a treatment(s), *n* (%) | 0 | 0 | 1 (4.3) | 0 | 1 (3.0) | 1 (4.0) | 0 | 0 | 3 (2.7) |
| Decrease the current treatment(s), *n* (%) | 0 | 0 | 1 (4.3) | 0 | 3 (9.1) | 1 (4.0) | 0 | 1 (6.7) | 6 (5.5) |

**Table S3** Investigator therapeutic attitude at baseline by country – not-controlled patients

| **Investigator therapeutic attitude at baseline** | **Belgium (*n* = 3)** | **Brazil (*n* = 12)** | **France (*n* = 25)** | **Germany (*n* = 11)** | **Italy (*n* = 21)** | **The Netherlands (*n* = 12)** | **Spain (*n* = 8)** | **UK (*n* = 5)** | **USA (*n* = 8)** | **Total (*n* = 105)** |
| --- | --- | --- | --- | --- | --- | --- | --- | --- | --- | --- |
| Continue the current treatment (s) with no change/no treatment(s) initiation, *n* (%) | 2 (66.7) | 11 (91.7) | 6 (24.0) | 6 (54.5) | 6 (28.6) | 3 (25.0) | 3 (37.5) | 3 (60.0) | 2 (25.0) | 42 (40.0) |
| Intensify the current treatment(s)/initiate a treatment(s), *n* (%) | 1 (33.3) | 1 (8.3) | 19 (76.0) | 4 (36.4) | 14 (66.7) | 9 (75.0) | 5 (62.5) | 2 (40.0) | 6 (75.0) | 61 (58.1) |
| Other, *n* (%) | 0 | 0 | 0 | 1 (9.1) | 1 (4.8) | 0 | 0 | 0 | 0 | 2 (1.9) |

Other investigator therapeutic attitudes at baseline were ‘under evaluation for neurosurgery’ and ‘will initiate a treatment 3 months following surgery’.
